# Depression in Diarrhea-Predominant IBS Patients: Exploring the Link Between Gut Barrier Dysfunction and Erythrocyte Polyunsaturated Fatty Acid Levels

**DOI:** 10.3390/jcm14072483

**Published:** 2025-04-05

**Authors:** Michele Linsalata, Laura Prospero, Antonia Ignazzi, Giuseppe Riezzo, Benedetta D’Attoma, Domenica Mallardi, Francesco Goscilo, Maria Notarnicola, Valentina De Nunzio, Giuliano Pinto, Francesco Russo

**Affiliations:** 1 Functional Gastrointestinal Disorders Research Group, National Institute of Gastroenterology IRCCS “S. de Bellis”, 70013 Castellana Grotte, Italy; michele.linsalata@irccsdebellis.it (M.L.); laura.prospero@irccsdebellis.it (L.P.); antonia.ignazzi@irccsdebellis.it (A.I.); giuseppe.riezzo@irccsdebellis.it (G.R.); benedetta.dattoma@irccsdebellis.it (B.D.); domenica.mallardi@irccsdebellis.it (D.M.); francesco.goscilo@irccsdebellis.it (F.G.); 2 Laboratory of Nutritional Biochemistry, National Institute of Gastroenterology IRCCS “S. de Bellis”, 70013 Castellana Grotte, Italy; maria.notarnicola@irccsdebellis.it (M.N.); valentina.denunzio@irccsdebellis.it (V.D.N.); giuliano.pinto@irccsdebellis.it (G.P.)

**Keywords:** intestinal permeability, polyunsaturated fatty acids, irritable bowel syndrome, depression, omega-3 polyunsaturated fatty acids, inflammation

## Abstract

**Background**: Patients with irritable bowel syndrome (IBS) often experience comorbid psychological conditions, notably depression and anxiety. Evidence suggests that these conditions are linked to gut barrier dysfunction, dysbiosis, and chronic inflammation. All these factors are central to IBS pathophysiology and mood disturbances. Polyunsaturated fatty acids (PUFAs) play crucial roles in modulating inflammation and depression. This study examined the associations among intestinal permeability, PUFA profiles, low-grade inflammation, and depression severity in IBS patients with diarrhea (IBS-D). **Methods**: Forty-three IBS-D patients (7 men, 36 women; 44.56 ± 1.52 years) were categorized into depressed (IBS-D(d+)) and non-depressed (IBS-D(d−)) groups according to scores on the depression subscale of the Symptom Checklist-90-Revised (SCL-90-R). Biomarkers of small intestinal permeability (s-IP) were assessed in urine and blood, alongside erythrocyte membrane PUFA composition, dysbiosis, and inflammation indices. **Results**: IBS-D (d+) patients exhibited elevated s-IP and altered PUFA metabolism compared to their IBS-D (d−) counterparts. Additionally, in the first group, omega-3 PUFA concentrations inversely correlated with s-IP biomarkers, while the omega-6/omega-3 ratio showed a positive correlation. Moreover, depression severity is significantly associated with s-IP markers and omega-3 PUFA levels. Lastly, IBS-D (d+) patients exhibited higher levels of dysbiosis and pro-inflammatory cytokines than IBS-D (d−) patients. **Conclusions**: These findings highlight the interplay between intestinal barrier integrity and PUFA metabolism in IBS-D patients with depression, suggesting that s-IP markers and erythrocyte PUFA profiles could represent novel therapeutic targets for managing depression in this population. This study was registered on ClinicalTrials.gov (NCT03423069), with a date of registration of 30 January 2018.

## 1. Introduction

Irritable bowel syndrome (IBS) is one of the most common functional gastrointestinal (GI) disorders and is estimated to affect about 1 in 10 people globally. Studies have indicated that IBS affects people all over the world. Still, the prevalence can vary widely from around 7.0% in people in Southeast Asia and the Middle East to between 11.8 and 14.0% in people in North America, Northern Europe, and Australia, and between 15.0 and 21.0% in people in Southern Europe, Africa, and South America [1]. The variation is undoubtedly due to methodological differences between the studies, as well as differences in pathophysiology and potential risk factors for IBS, including genetics, gastrointestinal infections, varied diets, gut microbiomes, and psychological comorbidities. All these factors may act differently depending on the geographical context.

IBS is characterized by persistent abdominal pain or discomfort commonly associated with altered bowel habits, including changes in defecation frequency and stool form [2]. According to the Rome IV criteria and the Bristol Stool Form Scale, IBS is classified into several predominant subtypes: IBS-D (diarrhea-predominant), IBS-C (constipation-predominant), IBS-M (mixed type), and IBS-U (unclassified). This classification system enables clinicians to tailor treatment strategies to the specific subtype, thereby enhancing patient outcomes [3].

IBS-D is the most common subtype, accounting for 37–62% of all IBS cases. The development of IBS-D is influenced by multiple factors, including diet, disruptions in intestinal flora, compromised intestinal barrier function, stress, and psychological factors [4].

There is growing evidence indicating that patients with IBS-D often experience comorbid psychological conditions, such as depression and anxiety, which can significantly impact their quality of life (QoL), highlighting the need for comprehensive treatment approaches that address both physical and mental health aspects [5]. For example, 50% to 60% of IBS patients have been found to have significant psychosocial issues [6]. Additionally, according to some studies, between 20% and 40% of people with IBS exhibit depressive symptoms [7].

However, the exact pathophysiological mechanisms linking these conditions remain poorly understood. Furthermore, conventional antidepressant therapies are often ineffective in this population, underscoring the need for a deeper understanding of the mechanisms underlying depression in IBS-D to improve management strategies for this functional disorder [8].

Over the past two decades, research on the brain–gut–microbiota axis has gained significant momentum, particularly in the context of mental health disorders [9]. Studies have shown that anxiety and depression are associated with gut dysbiosis, where microbiota secrete lipopolysaccharide (LPS) endotoxins into the bloodstream. Empirical evidence suggests that LPS and peptidoglycan induce activation of the hypothalamic–pituitary–adrenal axis and elicit innate immune responses by stimulating nucleotide-binding oligomerization domain-containing protein-1 [10]. This, combined with compromised gut barrier integrity, can lead to systemic manifestations, including effects on the brain [11]. In this context, the concept of “leaky gut syndrome” has garnered increasing attention. The intestinal barrier serves two primary functions: first, it facilitates the digestion and absorption of nutrients, and second, it tightly regulates the transport of antigens from the intestinal lumen into the submucosa, thereby maintaining a balance between immune tolerance and inflammatory responses [12]. When this barrier is compromised, increased small intestinal permeability (s-IP) can occur, potentially leading to visceral hypersensitivity and low-grade immune activation, particularly in IBS-D patients [13].

Chronic inflammation is believed to play a critical role in the pathophysiology of IBS-D and its associated mood disorders. Emerging research has also highlighted the crucial role of fatty acids (FAs) in modulating the balance between anti-inflammatory and pro-inflammatory responses [14]. Furthermore, FA levels are regarded as possible biological markers of depression, and changes in FAs metabolism may affect treatment outcomes for the condition [15]. Among FAs, polyunsaturated fatty acids (PUFAs), particularly omega-3 PUFAs and the omega-6/omega-3 PUFA ratio, have been extensively studied. Reduced levels of omega-3 PUFAs have been observed in the serum, plasma, and red blood cell membranes of patients with depression [16,17,18]. The lipid profile of erythrocyte membranes provides a systemic “snapshot” of nutritional status, reflecting the interplay of genetic, metabolic, and dietary factors [19]. Specifically, omega-3 PUFAs are essential components of neuronal cell membranes and play a vital role in various neurophysiological processes. They also serve as precursors to eicosanoids, which can reduce the levels of pro-inflammatory eicosanoids and cytokines associated with depression [20]. Moreover, initial evidence suggests that PUFAs may influence serotonin and dopamine signaling, providing a possible partial explanation of their beneficial effects on mood regulation in patients with IBS-D and depression [21,22,23].

Our previous work demonstrated a link between erythrocyte PUFA content and changes in intestinal barrier function in IBS-D patients. Elevated fecal zonulin levels, indicative of a compromised intestinal barrier, were associated with altered erythrocyte PUFA composition [24]. However, there is a lack of data on the relationship between PUFA levels and intestinal barrier markers in IBS-D patients with comorbid depression. We have hypothesized that a correlation exists between compromised intestinal permeability, alterations in PUFA composition, and the manifestation of depressive symptomatology in IBS-D patients.

Currently, several biomarkers are used to assess s-IP, including urinary levels of lactulose (Lac), mannitol (Man), and sucrose (Suc), as well as fecal zonulin and intestinal fatty acid-binding protein (I-FABP). Lac is a disaccharide that provides information on the paracellular pathway and tight junctions (TJs) integrity, while Man is a monosaccharide that is thought to reflect the transcellular route. An elevated Lac/Man ratio indicates s-IP dysfunction, while Suc levels reflect gastroduodenal permeability [25]. Zonulin, the only known physiological modulator of TJs, is critical in maintaining barrier function [26]. I-FABP belongs to a superfamily of proteins that regulate FAs uptake and transport into cells. When the intestinal epithelial barrier is damaged, I-FABP is released into the circulation, increasing plasma concentrations [27].

In this context, we conducted a study to examine the associations between these s-IP biomarkers, erythrocyte PUFA content, low-grade inflammation, and depression severity in patients with IBS-D. To further investigate these interactions, we assessed urinary indican (a marker of small intestinal dysbiosis) and circulating lipopolysaccharide (LPS) levels (a marker of bacterial translocation). Inflammatory status was evaluated by measuring interleukins (IL-6, IL-8, IL-10) and tumor necrosis factor-alpha (TNF-α).

Our research aimed to elucidate the underlying mechanisms contributing to IBS-D pathophysiology and identify potential therapeutic targets. Unlike previous research that primarily focused on the effects of PUFAs on individual symptoms, our approach evaluated GI and psychological outcomes. This integrative perspective aligns with the growing recognition that effective IBS-D management requires addressing both the physiological and psychological dimensions of the disorder.

## 2. Materials and Methods

### 2.1. Patient Recruitment

This study focused on patients with IBS-D, diagnosed according to the Rome IV criteria. These participants were recruited from the National Institute of Gastroenterology “S. de Bellis” Research Hospital in Castellana Grotte, Bari, Italy, between January 2022 and May 2022. Each participant underwent a comprehensive evaluation, including validated psychological and GI symptom questionnaires, a detailed physical examination, and biochemical testing. Blood samples were collected to assess complete blood count, liver function, C-reactive protein levels, and thyroid function. Stool samples were analyzed for culture, fecal parasites, and occult blood. To rule out organic diseases, all participants underwent gastroscopy and colonoscopy.

Inclusion criteria for IBS-D patients were as follows: (1) age over 18 years; (2) active IBS-D-like symptoms persisting for at least two weeks, with stool patterns consistent with those described by Schmulson et al. [28]; (3) a total IBS Severity Scoring System (IBS-SSS) score greater than 75; and (4) no dietary restrictions, particularly no gluten-free diet before the study. To ensure a homogeneous group, factors such as age, body mass index, alcohol consumption, smoking, and medication use were carefully evaluated. Exclusion criteria included pregnancy, constipation, giardiasis, post-infectious IBS, hepatic, renal, or cardiovascular diseases, metabolic or endocrine disorders, a history of SSRIs or other antidepressants, fever, strenuous physical activity, prior abdominal surgery, malignancy, secondary causes of intestinal atrophy, and recent use of IBS medications, antibiotics, probiotics, or other drugs known to cause abdominal pain within two weeks before the study. Celiac disease was ruled out using tissue transglutaminase (tTG) and anti-endomysial (EMA) antibody tests.

We next categorized IBS-D patients according to the presence or absence of depression IBS-D(d+) and IBS-D(d−), as measured by the depression subscale of the SCL-90-R.

Healthy controls (HCs) were recruited from the administrative staff of the institute and nursing students at the University of Bari, Italy. These individuals had no history of dyspepsia, GI disorders, metabolic, endocrine, or immune diseases, and were not taking any medications. Their health status was confirmed through interviews about diet, lifestyle, medical history, and a final physical examination. Inclusion criteria for HCs required negative EMA and tTG tests, normal blood glucose, HbA1c, lipid levels, blood pressure, and the absence of major mental illness, cancer, or pregnancy. Female participants, both IBS-D patients and HCs, were studied during the follicular phase of their menstrual cycle.

All participants provided written informed consent for blood and urine testing and clinical data collection. This study was registered on ClinicalTrials.gov (NCT03423069), with a date of registration of 30 January 2018. Approval was granted on December 18, 2017 by the Local Scientific Committee and the Institutional Ethics Committee of the IRCCS Oncological Hospital—John Paul II Cancer Institute, Bari, Italy (No. 274/E.V.).

### 2.2. IBS Severity Scoring System (IBS-SSS)

The symptom profile was assessed by the validated IBS-SSS questionnaire. This is a valuable instrument for quantifying IBS symptom severity across five key parameters using a visual analog scale [29]. These parameters include “abdominal pain intensity”, “abdominal pain frequency”, “abdominal bloating severity”, “dissatisfaction with bowel habits”, and “symptom impact on daily life”. Each parameter is rated on a scale from 0 to 100.

For four of the five items (abdominal pain intensity, bloating severity, bowel habit dissatisfaction, and symptom impact), patients select a point along a continuum, with the score determined by its distance from zero. The frequency of abdominal pain is reported as the percentage of days affected, multiplied by ten to align with the 0–100 scale. The overall IBS-SSS score is calculated by summing all five individual scores, resulting in a total score ranging from 0 to 500. Based on this total score, symptom severity is categorized as mild (75–175), moderate (>175–300), or severe (>300). Generally, scores below 75 indicate either healthy status or symptom remission.

### 2.3. Psychological Questionnaire

The Symptom Checklist-90-Revised (SCL-90-R) was used to evaluate the psychological profile. This questionnaire is one of the most popular and widely used self-report measures in psychopathology [30]. The SCL-90-R is a self-assessment questionnaire developed to provide a standardized measure of a person’s current psychological and psychopathological status. It is appropriate for normal or psychiatric populations, including adults and adolescents. This instrument examines nine primary dimensions (somatization, obsession-compulsion, interpersonal sensitivity, depression, anxiety, hostility, phobic anxiety, paranoid ideation, and psychoticism) as well as three global indices of disorder, of which we only considered the Global Severity Index (GSI), which measures the overall severity of the current disorder. After converting the raw scores into T scores, T scores of 63 or higher were considered indicative of clinically significant symptomatology [31]. The IBS-QoL is a self-administered quality-of-life (QoL) instrument designed for individuals with IBS. It evaluates the impact of IBS and its treatments through 34 items, which are summed and averaged to produce a total score. The tool also includes eight subscales: dysphoria, activity interference, body image, health concerns, food avoidance, social reactions, sexual function, and relationships. Lower scores on the IBS-QoL reflect a better quality of life. The raw scores are converted into a standardized scale from 0 to 100 [32].

The SF-36 is a concise questionnaire developed to assess patients’ overall health and quality of life, irrespective of their specific medical condition. It comprises eight subscales, with higher scores indicating better health status. The first three subscales focus on physical health, measuring physical functioning, role limitations due to physical problems, and bodily pain. The following two subscales (general health and vitality) evaluate overall well-being and energy levels. The final three subscales address mental and emotional health, including social functioning, role limitations due to emotional problems, and mental health. Additionally, the SF-36 includes a single unscaled item that tracks changes in the respondent’s health over the past year. The survey also allows for the calculation of two composite indices: the Physical Health Scale and the Mental Health Scale. These indices summarize the results of the eight subscales into two overarching scores. All scores are coded, aggregated, and transformed to a scale of 0 (worst possible health) to 100 (best possible health) [33].

### 2.4. Intestinal Barrier Function and Integrity Biomarkers

Patients and HCs underwent an assessment of s-IP through a sugar absorption test following an overnight fast [34]. Initially, urine samples were collected to check for naturally occurring sugars. Participants then ingested a solution containing 10 g of Lac, 5 g of Man, and 40 g of Suc dissolved in 100 mL of water. Urine samples were collected over five hours post-ingestion, with total volumes recorded. A 2 mL aliquot from each sample was frozen at −80 °C until further analysis. Urinary concentrations of Lac, Man, and Suc were quantified via chromatographic techniques, as described in prior studies [35]. The percentage of each sugar excreted (%Lac, %Man, %Suc) was determined based on the initial intake, and the Lac/Man ratio was subsequently calculated. A ratio exceeding 0.03 indicated altered s-IP [36].

Additionally, serum samples were obtained from IBS-D patients and HCs after a 12 h fast and stored at −80 °C until analysis. Stool samples were also collected and frozen at −80 °C within 12 h of collection. Zonulin levels in feces were measured using ELISA kits provided by Immunodiagnostik AG (Bensheim, Germany), with fecal concentrations below 107 ng/mL classified as normal according to the manufacturer’s specifications. Serum I-FABP was analyzed using ELISA kits from Thermo Fisher Scientific (Waltham, MA, USA).

### 2.5. Biomarkers of Intestinal Dysbiosis, Bacterial Translocation, and the Indices of Inflammation

Urinary indican concentrations were determined using a standard colorimetric assay kit (Indican Assay Kit, ABNOVA Corporation, Taipei, Taiwan) [37]. Serum LPS levels were measured using an ELISA kit from Cloud-Clone Corp. (Katy, TX, USA). Additionally, serum concentrations of IL-6, IL-8, IL-10, and TNF-α were analyzed using ELISA kits supplied by Elab Science Biotechnology Inc. (Houston, TX, USA).

### 2.6. PUFAs Analysis

To extract FAs and convert lipids into fatty acid methyl esters (FAMEs), an automated system called Robot LNG-R1 (Lipinutragen-Tecan, Bologna, Italy) was used. The process began with the collection of whole blood samples in EDTA tubes. The samples were centrifuged at 4000× *g* for 5 min at 6 °C to separate the components. Mature red blood cells, aged at least three months, were isolated based on density. After removing the plasma, the cells were lysed osmotically to release the membrane pellets, which were then used for phospholipid extraction. The extraction was performed using the Bligh and Dyer method [38]. Next, the organic layer containing chloroform was carefully separated and evaporated using a centrifugal evaporator (Thermo Fisher Scientific, Waltham, MA, USA). The lipids were then transesterified with potassium hydroxide to produce FAMEs. Once the transesterification was complete, the FAMEs were re-suspended in n-hexane for further analysis. The analysis was conducted using gas chromatography, equipped with an autosampler, a split/splitless injector, a flame ionization detector, and a hydrogen gas generator (Thermo Fisher Scientific, Milan, Italy). This setup followed previously described methods [39]. To quantify the FAMEs, we used a standard reference mixture (Supelco 37-Component FAMEs Mix, Sigma-Aldrich, Milan, Italy). PUFAs are expressed as the mean percentage of red blood cell membrane composition.

### 2.7. Statistical Analysis

All results are presented as mean ± SEM unless stated otherwise. Non-parametric tests were employed for statistical analysis to avoid making assumptions about normal distribution. Differences between two groups were evaluated using the Mann–Whitney test, while comparisons involving more than two groups were assessed with the Kruskal–Wallis test, followed by Dunn’s post hoc test for pairwise comparisons. Correlations were analyzed using the Spearman rank correlation test. A *p*-value of less than 0.05 was considered statistically significant.

A multiple linear regression analysis was performed using a stepwise regression procedure. In this model, depression was treated as the dependent variable, while biochemical parameters served as independent variables. The explained variance of the regression model was determined using the adjusted R-squared value and assessed with the F-test. To evaluate the contribution of each independent variable, t-values and their corresponding significance levels were calculated to determine whether the regression coefficients differed significantly from zero.

Statistical analyses were conducted using SigmaStat 11.0 (Systat Software, Inc., Chicago, IL, USA), and graphs were generated using GraphPad Prism 8 (GraphPad Software Inc., La Jolla, CA, USA).

## 3. Results

### 3.1. Study Groups’ Description and the Symptom Profile

**Figure 1:**
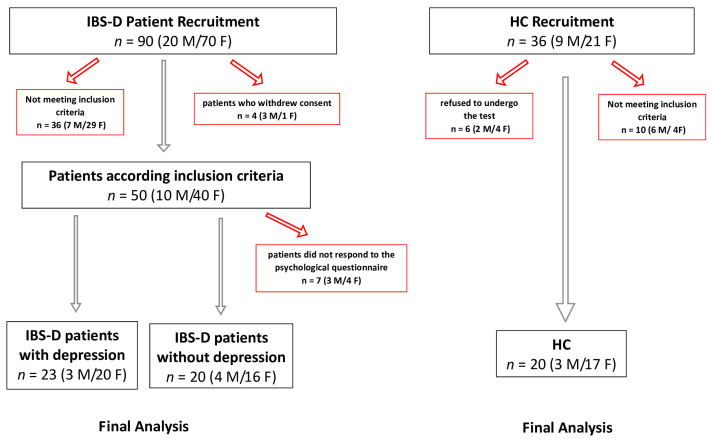
The study flowchart. IBS-D: diarrhea-predominant irritable bowel syndrome; HCs: healthy controls. Red arrows indicate patients who dropped out of the study. Black arrows indicate patients who completed the study.

Figure 1 reports the study flowchart detailing patient enrollment and analysis. Initially, 90 IBS-D patients (20 males and 70 females) were recruited. Following exclusions, 43 IBS-D patients and 20 HCs remained for the final analysis. Grouping the IBS-D patients according to scores on the depression subscale of the SCL-90-R, 23 patients (53.4%, 3 males and 20 females; mean age = 46.17 ± 1.98 years) had a depression cutoff score equal to or above 63, IBS-D(d+), whereas 20 patients (46.6%, 4 males and 16 females; mean age = 42.7 ± 2.31 years) had a cutoff score lower than 63, IBS-D(d−). All data collected for each individual patient are contained within the dataset reported in Supplementary Material S1.

Table 1 details the demographic, symptomatic, and psychological characteristics of both IBS-D patients and HC subjects. As expected, GI symptoms differed markedly between patients with IBS-D and HCs. In contrast, GI symptoms—based on both the total score and individual items of the IBS-SSS questionnaire—were similar between patients with IBS-D(d+) and IBS-D(d−).

In assessing psychological factors and QoL, significant differences emerged between IBS-D(d+) and IBS-D(d−) groups. Patients with IBS-D(d+) showed a significantly higher (*p* < 0.0001) score on the total IBS-QoL questionnaire score, demonstrating a worse perception of their QoL, than both IBS-D(d−) patients and HCs. Regarding the more purely psychopathological aspect investigated with the SCL-90-R, again the IBS-D(d+) had significantly higher pathological scores at the anxiety and somatization subscale, as well as at the GSI score, than the IBS-D(d−) and HCs. Moreover, on the Mental Health Scale of the SF-36, IBS-D(d+) patients reported significantly lower (*p* = 0.0003) scores compared to those with IBS-D(d−).

### 3.2. Biomarkers of Intestinal Barrier Function and Integrity

All participants, including IBS-D patients and HCs, underwent s-IP testing. The results revealed significant differences between the groups. IBS-D patients had a much higher %Lac than the controls (0.399 ± 0.062 vs. 0.175 ± 0.014, *p* < 0.0001). In contrast, the %Man was similar between IBS-D patients and HCs (13.19 ± 0.48 vs. 13.45 ± 0.61, *p* = 0.87). As a result, the Lac/Man ratio was significantly higher in IBS-D patients (0.031 ± 0.004) than in HCs (0.013 ± 0.001), *p* < 0.0001. Additionally, IBS-D patients exhibited a significantly higher %Suc excretion than HCs (0.241 ± 0.049 vs. 0.105 ± 0.013, *p* = 0.043).

Fecal zonulin and serum I-FABP levels were also significantly elevated in IBS-D patients. Fecal zonulin levels were 164.40 ± 12.54 ng/mL in IBS-D patients versus 98.95 ± 10.59 ng/mL in HCs (*p* = 0.0018). Similarly, serum I-FABP levels were higher in IBS-D patients (2.30 ± 0.31 ng/mL) than in HCs (1.44 ± 0.12 ng/mL), *p* = 0.040.

**Figure 2:**
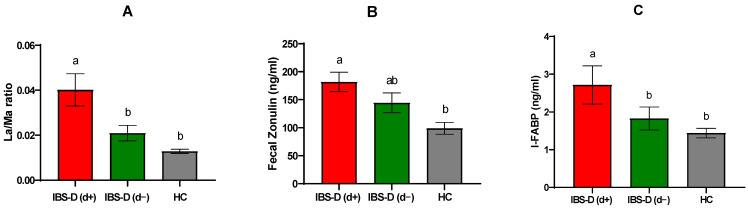
Urinary and serum markers of intestinal permeability in diarrhea-predominant irritable bowel syndrome (IBS-D) patients categorized according to depression and healthy controls (HCs). Lac/Man (%Lac to %Man) ratio (**A**), fecal zonulin (**B**), and intestinal fatty acid-binding protein (I-FABP) (**C**). IBS-D(d+): IBS-D patients with depression (cutoff score ≥ 63); IBS-D(d−): IBS-D without depression (cutoff score < 63). Data are expressed as mean ± SEM and analyzed by the Kruskal–Wallis test with Dunn’s multiple comparison test. Bars not sharing the same letter are significantly different (*p* < 0.05).

When IBS-D patients were further categorized based on the presence of depression symptoms, those with depression IBS-D (d+) exhibited even more pronounced differences. IBS-D(d+) patients had significantly higher %Lac excretion (0.494 ± 0.103) compared to both IBS-D(d−) (0.290 ± 0.056) and HCs (0.175 ± 0.014), *p* = 0.008 and *p* < 0.0001, respectively. While %Man excretion did not differ significantly among the groups, IBS-D(d+) patients showed slightly lower %Man excretion (12.67 ± 0.66) compared to IBS-D(d−) (13.78 ± 0.71) and HCs (13.45 ± 0.615). The Lac/Man ratio was also significantly higher in IBS-D(d+) patients (0.040 ± 0.007) compared to both IBS-D(d−) (0.021 ± 0.003) and HCs (0.013 ± 0.001), *p* = 0.004 and *p* < 0.0001, respectively (Figure 2A).

Gastroduodenal permeability, measured by %Suc excretion, did not differ significantly among the groups. However, IBS-D(d+) patients had higher %Suc excretion (0.276 ± 0.062) compared to IBS-D(d−) (0.202 ± 0.079) and HCs (0.105 ± 0.013). Fecal zonulin concentrations were also elevated in IBS-D(d+) patients (181.70 ± 17.25 ng/mL) compared to IBS-D(d−) (144.50 ± 17.66 ng/mL), reaching a statistically significant difference compared to HCs (98.95 ± 10.59 ng/mL) *p* = 0.0031 (Figure 2B). Circulating levels of I-FABP were significantly higher in IBS-D(d+) patients (2.718 ± 0.505 ng/mL) compared to both IBS-D(d−) (1.825 ± 0.306 ng/mL) and HCs (1.441 ± 0.123 ng/mL), *p* = 0.040 and *p* = 0.009, respectively (Figure 2C).

### 3.3. Biomarkers of Intestinal Dysbiosis, Bacterial Translocation, and the Indices of Inflammation

IBS-D patients exhibited significantly higher urinary indican levels than HCs (67.09 ± 5.23 mg/L vs. 50.75 ± 4.83 mg/L, *p* = 0.047). Serum LPS concentrations were also significantly elevated in IBS-D patients relative to the controls (0.058 ± 0.010 ng/mL vs. 0.037 ± 0.005 ng/mL, *p* = 0.039).

Regarding inflammatory markers, mean serum IL-6 levels were significantly increased in IBS-D patients compared to the HC group (6.04 ± 0.85 ng/mL vs. 4.05 ± 0.19 ng/mL; *p* < 0.0001). No significant difference was observed for IL-8 levels between IBS-D patients (4.64 ± 0.35 ng/mL) and HCs (4.77 ± 0.07 ng/mL). However, TNF-α concentrations were significantly higher in IBS-D patients than in HCs (3.49 ± 0.10 ng/mL vs. 3.30 ± 0.29 ng/mL; *p* = 0.004). Conversely, mean serum IL-10 levels were significantly lower in IBS-D patients than in HCs (2.87 ± 0.04 ng/mL vs. 3.46 ± 0.13 ng/mL; *p* < 0.0001).

**Figure 3:**
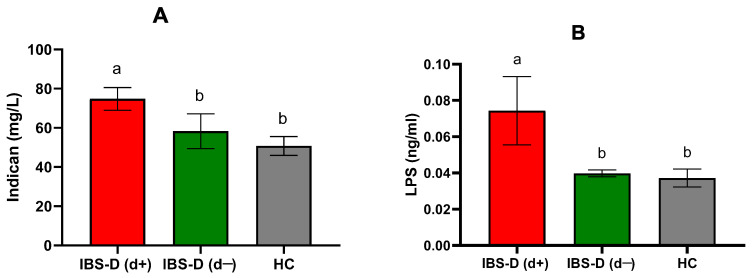
Urinary indican (**A**) and serum lipopolysaccharide (LPS) (**B**) levels in diarrhea-predominant irritable bowel syndrome (IBS-D) patients categorized according to depression and healthy controls (HCs). IBS-D(d+): IBS-D patients with depression (cutoff score ≥ 63); IBS-D(d−): IBS-D without depression (cutoff score < 63). Data are expressed as mean ± SEM and analyzed by the Kruskal–Wallis test with Dunn’s multiple comparison test. Bars not sharing the same letter are significantly different (*p* < 0.05).

When stratified by depression symptoms, urinary indican levels varied significantly among the groups. The IBS-D(d+) subgroup had significantly higher indican levels (74.78 ± 5.79 mg/L) than both the IBS-D(d−) subgroup (58.25 ± 8.83 mg/L, *p* = 0.045) and HCs (50.70 ± 4.83 mg/L, *p* = 0.012) (Figure 3A).

Serum LPS concentrations also differed significantly among the groups, with IBS-D(d+) patients displaying significantly higher levels (0.074 ± 0.018 ng/mL) compared to IBS-D(d−) (0.039 ± 0.001 ng/mL), *p* = 0.030, and HCs (0.037 ± 0.005 ng/mL), *p* = 0.007 (Figure 3B).

Regarding cytokines, IL-6 levels were significantly higher in IBS-D(d+) compared to IBS-D(d−) (*p* = 0.038) and HCs (*p* < 0.0001). Moreover, IL-6 concentrations in IBS-D(d−) were significantly elevated compared to HCs (*p* = 0.042). Similarly, TNF-α levels were significantly increased in IBS-D(d+) relative to IBS-D(d−) (*p* = 0.033) and HCs (*p* = 0.0007). Conversely, IL-10 serum levels were significantly lower in both IBS-D(d+) and IBS-D(d−) subgroups when compared to HCs (*p* < 0.0001 and *p* = 0.0008, respectively). No significant differences in IL-8 levels were observed among groups (Table 2).

### 3.4. PUFAs Profile

The overall group of IBS-D patients exhibited significantly higher (*p* < 0.0001) n-6 PUFA levels than HCs (28.68 ± 0.71 vs. 22.67 ± 0.53). Conversely, in IBS-D patients, n-3 PUFA levels were significantly lower (*p* = 0.004) compared to HCs (7.93 ± 0.52 vs. 9.62 ± 0.47). As a result, the n-6/n-3 PUFA ratio was markedly and significantly (*p* < 0.0001) elevated in IBS-D patients compared to HCs (4.55 ± 0.48 vs. 2.51 ± 0.18).

**Figure 4:**
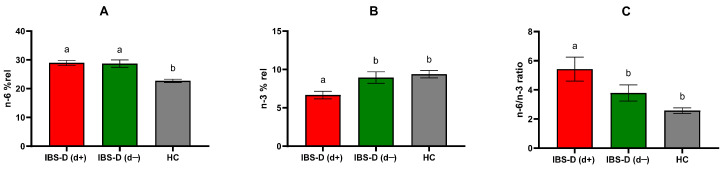
Mean percentage of red blood cell membrane n-6 (**A**), n-3 (**B**) and n-6/n-3 ratio (**C**) in diarrhea-predominant irritable bowel syndrome (IBS-D) patients categorized according to depression and healthy controls (HCs). IBS-D(d+): IBS-D patients with depression (cutoff score ≥ 63); IBS-D(d−): IBS-D without depression (cutoff score < 63); n-6: omega-6 polyunsaturated fatty acids, n-3: omega-3 polyunsaturated fatty acids. Data are expressed as mean ± SEM and analyzed by the Kruskal–Wallis test with Dunn’s multiple comparison test. Bars not sharing the same letter differ significantly (*p* < 0.05).

When stratified based on depressive symptoms, IBS-D(d+) patients had significantly higher (*p* < 0.0001) n-6 PUFA levels (28.94 ± 0.86) compared to HCs (22.67 ± 0.53). Notably, IBS-D(d+) subjects had significantly lower n-3 PUFA levels (6.69 ± 0.47) than both IBS-D(d−) (9.35 ± 0.88, *p* = 0.022) and the controls (9.62 ± 0.47, *p* = 0.0006). The n-6/n-3 PUFA ratio was significantly higher in IBS-D(d+) patients (5.29 ± 0.76) compared to both IBS-D(d−) (3.70 ± 0.51) and HCs (2.51 ± 0.18), *p* = 0.038 and *p* < 0.0001, respectively (Figure 4). All the PUFA values were expressed as a mean percentage of red blood cell membrane PUFAs (% rel).

### 3.5. Correlations and Regression Analyses

In the IBS-D(d+) subgroup, n-3 PUFAs exhibited a negative correlation with %Lac (r = −0.463, *p* = 0.026) and %Suc (r = −0.457, *p* = 0.028). Conversely, the n-6/n-3 PUFA ratio was positively correlated with %Lac (r = 0.495, *p* = 0.016) and fecal zonulin (r = 0.449, *p* = 0.031). No significant correlations were observed in the IBS-D(d−) subgroup.

Regression analysis showed that the level of depression calculated in IBS-D patients could be significantly predicted by a linear combination of the variables considered in a stepwise procedure. Specifically, n-3 PUFAs contributed negatively, whereas %Lac contributed positively to the model (F = 4.698; df = 3; *p* = 0.007; adjusted R^2^ = 0.209) (Table 3). These results suggest that specific biochemical parameters may play a role in influencing depression severity (Table 3).

## 4. Discussion

The IBS-D pathogenesis involves multiple factors and mechanisms, including dysmotility, visceral hypersensitivity, and abnormal brain–gut interactions [40]. Data in the literature indicate that depression is a frequent comorbidity of IBS-D and that common pathophysiological mechanisms may underline both conditions [6,41].

Epidemiological studies indicate that depression is a common comorbidity in patients with IBS. A meta-analysis reported that the prevalence of depressive symptoms among IBS patients is approximately 28.8% [42]. In addition, it has been shown that the presence and severity of depression are strongly associated with the prevalence of intestinal and extra-intestinal symptoms in patients with IBS [43]. These findings underscore the importance of routine psychological assessment and integrated care approaches in managing IBS-D patients.

While different theories plausibly explain the symptoms of depressive states [44], there is a lack of data on the molecular mechanisms underlying depression, particularly in multifactorial diseases such as IBS-D. This investigation underscores the significant contribution of the GI tract to the manifestation of depressive symptomatology in IBS-D patients. Specifically, the in vivo data presented elucidate a strong correlation between sympathetic nervous system activity, s-IP, and PUFA metabolism. The findings of this study offer novel insights into the complex interplay between the gut and the central nervous system in this patient population. The primary and novel findings of this study were **the demonstration of** increased s-IP and concomitant alterations in PUFA metabolism in IBS-D(d+) compared to IBS-D(d−). Furthermore, in the first group, omega-3 PUFA concentrations inversely correlated with s-IP biomarkers, while the omega-6/omega-3 ratio showed a positive correlation. To our knowledge, this is the first study to investigate the interaction between altered IP and erythrocyte PUFA levels in IBS-D patients with depressive symptoms. We found that s-IP markers, such as the Lac/Man ratio and I-FABP levels, were significantly increased in IBS-D(d+) patients compared to IBS-D(d−) ones and HCs. This suggests a key role for intestinal barrier dysfunction in the pathogenesis of IBS-D with comorbid depression. These findings indicate that biochemical and pathophysiological parameters, such as PUFA levels and s-IP markers, may play a role in influencing depression severity in IBS-D patients. In addition, they align with recent studies, such as that by Stevens et al. [45], which reported significantly elevated plasma I-FABP levels in patients with anxiety and depression compared to healthy subjects.

It is well established that psychiatric disorders are often accompanied by dysbiosis and bacterial translocation, particularly when the gut barrier is compromised [46,47,48]. For instance, Calarge et al. [49] observed increased intestinal permeability associated with bacterial translocation in depressed adolescents. Similarly, Alonso et al. [50] demonstrated that the stress-induced disruption of intestinal permeability in humans was associated with the increased transport of macromolecules and LPS. In line with these findings, our study revealed significantly higher levels of indican and LPS in IBS-D(d+) patients compared to IBS-D(d−) and HCs. It has been hypothesized that dysbiosis increases intestinal barrier permeability, leading to a “leaky gut” characterized by elevated circulating LPS levels, an endotoxin derived from the cell walls of Gram-negative bacteria. These changes trigger the production of pro-inflammatory cytokines, which in turn reduce neurotransmitter levels, contributing to the development of depression [51]. This “leaky gut hypothesis” may partially explain the association between inflammation and depression, as depressive symptoms are also linked to an inflammatory response. In this regard, our study also observed higher levels of pro-inflammatory cytokines, such as IL-6 and TNF-α, in IBS-D(d+) patients compared to IBS-D(d−) patients.

PUFAs are another critical factor in inflammatory processes and depression. Abnormalities in PUFA metabolism are known to induce chronic low-level systemic inflammation and are implicated in the progression of depression [17]. Specifically, considerable attention has been devoted to studying n-3 PUFA levels and the n-6/n-3 PUFA ratio, which are often reduced in the serum/plasma and red blood cell membranes of patients with depression [52]. Interestingly, in our study, when patients were categorized based on depressive symptoms, the IBS-D(d+) subgroup exhibited significantly lower n-3 PUFA levels and a higher n-6/n-3 PUFA ratio than the IBS-D(d−) subgroup and HCs. Two main neurophysiological mechanisms have been proposed to explain the link between PUFAs and depression. First, a growing body of evidence supports the connection between depression and the production of pro-inflammatory cytokines [53]. Omega-3 PUFAs are well-documented inhibitors of pro-inflammatory cytokines and inflammatory eicosanoids, which appear to reduce epithelial cell responses to inflammatory stimuli and improve inflammation-related outcomes [54]. Conversely, n-6 PUFAs can activate adipocyte inflammatory responses, leading to the accumulation of reactive oxygen species and pro-inflammatory factors.

A second potential mechanism involves the role of n-3 PUFAs in maintaining membrane integrity and fluidity, which is crucial for neurotransmitter binding and intracellular signaling [55]. Previous in vitro studies and rodent models have demonstrated that n-3 PUFAs influence TJs protein connections, preventing the uncontrolled paracellular permeability of substances such as LPS into the bloodstream [56]. Both in vitro and in vivo investigations have demonstrated that n-3 PUFAs modulate TJs proteins, specifically occludin and zonula occludens-1, which are crucial for maintaining effective intercellular connections and preventing uncontrolled paracellular permeability. In a human vascular endothelial cell line, eicosapentaenoic acid, which also exhibits antineoplastic properties, has been observed to regulate occludin expression, thereby contributing to the modification of the transendothelial electrical resistance of these cells. Additionally, n-3 PUFAs activate G-protein coupled receptor 120, eliciting anti-inflammatory responses and enhancing TJs’ stability [57,58]. Recent human trials have demonstrated that dietary n-3 PUFAs are linked to improvements in impaired intestinal barrier integrity [59]. Our previous work identified a strong link between impaired intestinal barrier function and altered erythrocyte PUFA composition in IBS-D patients [24]. Clinical observations indicate a frequent comorbidity of psychological disorders, specifically depression, in patients diagnosed with IBS-D. Nevertheless, there exists a paucity of data concerning the correlation between intestinal barrier markers and PUFA concentrations within the IBS-D patient population exhibiting comorbid depressive symptomatology. In the present study of IBS-D(d+) patients, n-3 PUFA levels showed a negative correlation with % Lac, a marker of intestinal paracellular permeability, while the n-6/n-3 ratio exhibited a positive correlation with %Lac and with fecal zonulin, a physiological modulator of TJs. These correlations were not observed in the IBS-D(d−) group. Moreover, multiple regression analysis indicated that depression levels were mainly associated with %Lac and n-3 PUFA concentrations but not n-6 PUFA levels.

This study suggests that in patients with IBS-D, depression may be associated with alterations in the PUFA profile. Specifically, low levels of n-3 PUFAs may contribute to both (1) impaired intestinal barrier function, leading to the increased translocation of macromolecules and LPS into the systemic circulation, and (2) the development of a systemic inflammatory state, potentially predisposing individuals to depression.

Inflammatory bowel disease (IBD) is another prevalent GI disorder. Notably, both IBS-D and IBD exhibit overlapping clinical manifestations and share common pathogenic mechanisms and etiological factors, including the regulation of the brain–gut axis and alterations in lipidomic profiles. A documented reduction in n-3 docosahexaenoic acid (DHA) is indicative of a dysregulation between anti-inflammatory and pro-inflammatory mediators, such as arachidonic acid (ARA). Recent research has demonstrated a significant decrease in DHA levels within patients with ulcerative colitis in remission exhibiting IBS-like symptomatology (UCR-IBS) [60]. Conversely, linolenic acid, ARA, and the omega-6/omega-3 PUFA ratio were significantly elevated in both the UCR-IBS and IBS-D patient cohorts. These observed PUFA imbalances suggest a potential shared pathophysiological mechanism within psychological tissues, and the activation of inflammatory pathways in both IBS-D and UCR-IBS [60].

The present findings underscore the close relationship between changes in intestinal barrier function and PUFA metabolism in IBS-D patients with depression, suggesting that s-IP markers and erythrocyte membrane PUFA levels could serve as novel therapeutic targets for managing depression in this population.

While the relationship between depression and altered intestinal barrier is well documented [61], the association with PUFAs in IBS-D patients represents a novel finding. Among the biological properties of n-3 PUFAs, their unique role in structural changes at the brain level is particularly noteworthy [62]. This highlights the need for further research to better understand how dietary adjustments in n-3 PUFA intake, especially in Western countries like Italy, could benefit specific populations, such as IBS-D patients.

Recent studies suggest that the potential clinical benefits of dietary PUFA intake have expanded beyond cardiovascular diseases to include rheumatological conditions, chronic kidney disease, inflammatory pulmonary diseases, and depression. Additionally, there is growing interest in the potential of PUFAs as a treatment for several GI conditions, such as colorectal cancer, functional gastrointestinal disorders, especially IBS, and IBD. The administered dosages and the particulars of the patient cohort chosen for the study appear to significantly influence the observed effects [63].

These results are innovative for several reasons. First, they provide a mechanistic view of depression–IBS-D comorbidity, going beyond the simple observation of their coexistence. Additionally, they identify gut barrier dysfunction and erythrocyte PUFA levels as potential therapeutic targets for the treatment of depression in patients with IBS-D.

This study has some limitations. The sample size was relatively small, limiting the capacity to draw definitive conclusions about the molecular mechanisms linking PUFA levels and intestinal barrier dysfunction in IBS-D patients with depression. Therefore, further in vitro and in vivo studies are needed to explore the impact of erythrocyte PUFA levels and intestinal barrier markers on the prevention, diagnosis, treatment, and prognosis of depression in IBS-D patients. Additionally, the suggested fermentative dysbiosis, as indicated by urinary indican levels, was not supported by more comprehensive methods, such as molecular analysis of the 16S rRNA gene, to characterize bacterial populations in the GI tract. Finally, patients did not observe any dietary restrictions prior to the study, and there was no assessment of dietary PUFA intake.

Future research should meticulously delineate the underlying pathophysiological mechanisms and the putative clinical efficacy of PUFA dietary supplementation. Specifically, clinical trials are warranted to comparatively assess the impact of PUFA intervention on the symptomatic presentation of IBS patients, juxtaposed against established dietary modalities, including fiber supplementation, probiotic administration, and carbohydrate alternative regimens. In addition, they should explore the efficacy of interventions involving PUFAs supplementation aimed at restoring gut integrity and optimizing omega-3 levels to improve both gut and depressive symptoms.

## 5. Conclusions

Our findings highlight the gut as a potential new target for managing depression, particularly in individuals with GI disorders such as IBS-D. This methodological framework is a component of a comprehensive therapeutic paradigm that integrates dietary modifications, pharmacological interventions, and psychological strategies to facilitate a personalized treatment regimen for functional disorders. Given the endogenous non-synthesizability of PUFAs and the substantial body of evidence supporting their therapeutic efficacy in various diseases, their dietary inclusion warrants consideration. This is the first study to analyze s-IP and erythrocyte PUFA content in IBS-D patients with depression, providing a link between sympathetic nervous system activation and increased s-IP, as well as altered PUFA metabolism. These changes likely contribute to the autonomic symptoms of depression, offering new insights into the complex interplay between the gut and the brain in this population.

## Figures and Tables

**Table 1:** The demographic, symptomatic, and psychological characteristics of IBS-D patients and HC subjects.

	IBS-D(d+) n = 23	IBS-D (d−) n = 20	HCs n = 20	*p*-Value
Demographics				
Sex(F/M)	20/3	16/4	17/3	ns
Age (years)	46.17 ± 1.98	42.7 ± 2.31	40.2 ± 1.84	ns
BMI (Kg/m^2^)	25.38 ± 0.98	25.19 ± 1.01	24.63 ± 0.97	ns
IBS-SSS				
Total score	277.0 ± 16.86 **^a^**	262.1 ± 19.79 **^a^**	47.2 ± 11.41 ^b^	*p* < 0.0001
Abdominal pain intensity	48.0 ± 4.84 ^a^	45.8 ± 5.47 ^a^	3.0 ± 1.75 ^b^	*p* < 0.0001
Abdominal pain frequency (days)	50.0 ± 6.16 ^a^	43.0 ± 6.61 ^a^	4.0 ± 2.66 ^b^	*p* < 0.0001
Abdominal bloating severity	54.3 ± 3.89 ^a^	57.7 ± 6.16 ^a^	3.0 ± 2.10 ^b^	*p* < 0.0001
Dissatisfaction with Bowel habits	64.2 ± 5.33 ^a^	64.2 ± 5.27 ^a^	28.0 ± 5.74 ^b^	*p* < 0.0001
Symptom impact on daily life	60.5 ± 4.35 ^a^	51.5 ± 5.64 ^a^	9.25 ± 2.72 ^b^	*p* < 0.0001
SF-36				
Physical health	45.0 ± 1.58 ^a^	47.4 ±1.69 ^a^	58.8 ± 1.08 ^b^	*p* < 0.0001
Mental health	26.2 ± 2.93 ^a^	40.5 ± 2.97 ^b^	43.3± 3.11 ^b^	*p* = 0.0003
IBS-QoL				
Total score	39.5 ± 3.44 **^a^**	21.18 ± 2.69 **^b^**	3.6 ± 1.23 **^c^**	*p* < 0.0001
SCL-90-R				
GSI (T scores)	80.6 ± 2.95 ^a^	51.7 ±1.98 ^b^	48.7 ± 2.68 ^b^	*p* < 0.0001
Somatization (T scores)	74.5 ± 2.96 ^a^	60.4 ± 3.43 ^b^	48.7 ± 2.15 ^b^	*p* < 0.0001
Anxiety (T score)	75.3 ± 3.63 ^a^	49.7 ± 2.23 ^b^	46.6 ± 2.35 ^b^	*p* < 0.0001

Abbreviations. HCs: healthy controls; IBS: irritable bowel syndrome; IBS-D: diarrhea-predominant irritable bowel syndrome; IBS-D(d+): IBS-D patients with depression (cutoff score ≥ 63); IBS-D(d−): IBS-D without depression (cutoff score < 63); IBS-SSS: IBS Severity Scoring System; QoL: quality of life. Data are expressed as mean ± SEM and analyzed by the Kruskal–Wallis test with Dunn’s multiple comparison test. Differences were considered significant at *p* < 0.05. Values not sharing the same letter are significantly different; ns: not statistically significant.

**Table 2:** Indices of inflammation in IBS-D subgroups and HCs.

	IBS-D (d+) (n = 23)	IBS-D (d−) (n = 20)	HCs (n = 20)	*p*-Value
IL-6 (ng/mL)	7.26 ± 1.55 ^a^	4.65 ± 0.19 ^b^	4.05 ± 0.19 ^c^	<0.0001
IL-8 (ng/mL)	4.33 ± 0.18 ^a^	5.00 ± 0.73 ^a^	4.77 ± 0.07 ^a^	n.s.
IL-10 (ng/mL)	2.82 ± 0.05 ^a^	2.92 ± 0.06 ^a^	3.46 ± 0.12 ^b^	<0.0001
TNF-α (ng/mL)	3.73 ± 0.14 ^a^	3.20 ± 0.11 ^b^	3.30 ± 0.29 ^b^	*p* = 0.0007

IBS-D: diarrhea-predominant irritable bowel syndrome; HCs: healthy controls; IBS-D(d+): IBS-D patients with depression (cutoff score ≥ 63); IBS-D(d−): IBS-D without depression (cutoff score < 63); IL-6, IL-8, and IL-10; interleukins 6, 8, and 10; TNF-α: tumor necrosis factor. n.s.: not significant. Data are expressed as mean ± SEM and analyzed by the Kruskal–Wallis test with Dunn’s multiple comparison test. Differences were considered significant at *p* < 0.05. Different superscripts differed significantly.

**Table 3:** Regression analysis between the level of depression and biochemical variables in IBS-D patients.

Parameters	(β)	Std. Error (β)	*p*	95% CI
%Lac	17.525	6.434	0.010	−0.402–1.800
n-3 PUFAs	−1.585	0.767	0.046	−3.088–0.081
n-6 PUFAs	0.699	0.562	0.221	−0.402–1.800

A linear regression analysis was performed considering the level of depression as the dependent variable and biochemical parameters as the independent variables. %Lac = % lactulose; n-3 PUFA: omega-3 polyunsaturated fatty acids, n-6 PUFA: omega-6 polyunsaturated fatty acids.

## Data Availability

The datasets used and/or analyzed during the current study are available as Supplementary Materials.

## Supplementary Materials

The following supporting information can be downloaded at: https://www.mdpi.com/article/10.3390/jcm14072483/s1, Dataset S1: raw data.xlsx.

## Abbreviations

The following abbreviations are used in this manuscript:

IBS	Irritable bowel syndrome
IBS-D	Diarrhea-predominant irritable bowel syndrome
PUFAs	Polyunsaturated fatty acids
s-IP	Small intestinal permeability
GI	Gastrointestinal
QoL	Quality of life
LPS	Lipopolysaccharide
TJs	Tight junctions
FAs	Fatty acids
n-3 PUFAs	Omega-3 polyunsaturated fatty acids
n-6 PUFAs	Omega-6 polyunsaturated fatty acids
IBS-C	Constipation-predominant irritable bowel syndrome
IBS-M	Mixed-type irritable bowel syndrome
IBS-U	Unclassified irritable bowel syndrome
Lac	Lactulose
Man	Mannitol
Suc	Sucrose
IBS-SSS	IBS Severity Scoring System
GSI	Global Severity Index
IBS-QoL	IBS Quality of Life Questionnaire
SF-36	Short Form Health Survey (36 items)
EMAs	Anti-endomysial antibodies
tTG	Tissue transglutaminase
EDTA	Ethylenediaminetetraacetic acid
FAMEs	Fatty acid methyl esters
I-FABP	Intestinal fatty acid-binding protein
IL-6	Interleukin-6
IL-8	Interleukin-8
IL-10	Interleukin-10
TNF-α	Tumor necrosis factor-alpha
HCs	Healthy controls
ELISA	Enzyme-linked immunosorbent assay
HbA1c	Glycated hemoglobin
SCL-90-R	Symptom Checklist-90-Revised
CFUs	Colony-forming units
FAMEs Mix	Fatty acid methyl esters mixture
IBD	Inflammatory bowel disease
DHA	Docosahexaenoic acid
ARA	Arachidonic acid
UCR-IBS	Ulcerative colitis in remission exhibiting IBS-like symptomatology
